# Polo-like kinase 3 and phosphoT273 caspase-8 are associated with improved local tumor control and survival in patients with anal carcinoma treated with concomitant chemoradiotherapy

**DOI:** 10.18632/oncotarget.10801

**Published:** 2016-07-23

**Authors:** Franz Rödel, Daniel Martin, Christina Helmke, Panagiotis Balermpas, Emmanouil Fokas, Ulrike Wieland, Margret Rave-Fränk, Julia Kitz, Yves Matthess, Monika Raab, Klaus Strebhardt, Claus Rödel

**Affiliations:** ^1^ Department of Radiotherapy and Oncology, Goethe-University, Frankfurt am Main, Germany; ^2^ Department of Gynecology, Goethe-University, Frankfurt am Main, Germany; ^3^ Institute of Virology, National Reference Centre for Papilloma- and Polyomaviruses, University of Cologne, Cologne, Germany; ^4^ Department of Radiotherapy and Radiation Oncology, University Medical Center Göttingen, Göttingen, Germany; ^5^ Department of Pathology, University Medical Center Göttingen, Göttingen, Germany; ^6^ German Cancer Research Center (DKFZ), Heidelberg, Germany; ^7^ German Cancer Consortium (DKTK) partnersite: Frankfurt, Heidelberg, Germany

**Keywords:** anal carcinoma, polo-like kinase 3, caspase-8, chemoradiotherapy, local control

## Abstract

We have recently shown that caspase-8 is a new substrate of Polo-like kinase 3 (Plk3) that phosphorylates the protein on residue T273 thereby promoting its pro-apoptotic function. In the present study we aimed to investigate the clinical relevance of Plk3 expression and phosphorylation of caspase-8 at T273 in patients with anal squamous cell carcinoma (SSC) treated with 5-fluorouracil and mitomycin C-based chemoradiotherapy (CRT). Immunohistochemical detection of the markers was performed in pretreatment biopsy specimens of 95 patients and was correlated with clinical/histopathologic characteristics including HPV-16 virus load/p16^INK4a^ expression and cumulative incidence of local and distant failure, cancer specific survival (CSS), and overall survival (OS). We observed significant positive correlations between Plk3 expression, pT273 caspase-8 signal, and levels of HPV-16 virus DNA load/p16^INK4a^ detection. Patients with high scores of Plk3 and pT273 caspase-8 showed increased local control (*p* = 0.011; *p* = 0.001), increased CSS (*p* = 0.011; *p* = 0.013) and OS (*p* = 0.024; *p* = 0.001), while the levels of pT273 caspase-8 were significantly associated (*p* = 0.033) with distant metastases. In multivariate analyses Plk3 expression remained significant for local failure (*p* = 0.018), CSS (*p* = 0.016) and OS (*p* = 0.023). Moreover, a combined HPV16 DNA load and Plk3 or pT273 caspase-8 variable revealed a significant correlation to decreased local failure (*p* = 0.001; *p* = 0.009), increased CSS (*p* = 0.016; *p* = 0.023) and OS (*p* = 0.003; *p* = 0.003). In conclusion these data indicate that elevated levels of Plk3 and pT273 caspase-8 are correlated with favorable clinical outcome in patients with anal SCC treated with concomitant CRT.

## INTRODUCTION

Polo-like-kinases (Plks) are master regulators of cell cycle progression, entry into mitosis, DNA replication and checkpoint regulation [1–3]. Within the Plk family, five members (Plk1, Plk2/Snk, Plk3/Fnk/Prk, Plk4/Sak and Plk5) are molecularly characterized by a canonical serine/threonine kinase domain located within the N-terminal part and a unique regulatory C-terminal polo-box domain (PBD) that is involved in the regulation of kinase activity, cellular localization and substrate recognition [4]. Moreover, malignant cells have dysregulated Plk activity with enhanced proliferation, migration, invasion and resistance to apoptotic cell death [5–7] that make Plk family members attractive targets for a molecular anti-cancer therapy. Various ATP-competitive small molecule Plk1 inhibitors successfully entered the clinics including BI 6727 (Volasertib^®^) and demonstrated survival benefit in patients with acute myeloid leukemia [8]. These drugs, however, also inhibit the activity of the Plk1-related family members Plk2 and Plk3 [9] indicating the necessity for a careful consideration of the role of these proteins in oncogenesis and treatment response.

While biological and clinical research highly focused on Plk1, the role of Plk3 is less investigated. Plk3, identified and cloned in our lab [10], displays divergent functional properties in terms of regulation during the cell cycle and response to growth factors and genotoxic stress [11–13]. Plk3 kinase activity is rapidly increased upon oxidative stress or DNA damage in an Ataxia telangiectasia mutated (ATM) kinase-dependent manner resulting in phosphorylation of the tumor suppressor TP53 and checkpoint kinase 2 (Chk2), linking DNA damage to cell cycle arrest and apoptosis [3, 14]. We have recently identified Plk3 as a novel interaction partner of the death receptor CD95 and caspase-8 as a new substrate of Plk3 that phosphorylates pro-caspase-8 on residue T273, thereby promoting its pro-apoptotic function upon CD95 or related apoptosis-inducing ligand (TRAIL) stimulation [15]. As caspase-8 is a key element of the extrinsic death pathway activated by death ligand/receptor mediated signaling [16], our observation implicates caspase-8 T273 phosphorylation as a novel mechanism to promote apoptotic signaling. Interestingly, controversial findings have been reported with regard to the prognostic impact of Plk3. Indeed, Plk3 has a tumor suppressor role in hepatocellular (HCC) and head and neck squamous cell carcinoma (HNSCC) [17–19], while overexpression was correlated with shortened relapse-free survival time in breast and ovarian cancer [20, 21].

In the present study we aimed to investigate the clinical relevance of Plk3 expression and caspase-8 phosphorylation at T273 in patients uniformly treated by standard CRT for anal SCC. Our data demonstrate that low initial Plk3 and pT273 caspase-8 levels were associated with low HPV16 DNA and p16^INK4a^ expression and with an increased risk of local failure and decreased survival, indicating that these tumors may represent a subpopulation of anal SCC at risk that may require intensified treatment strategies.

## RESULTS

**Figure 1:**
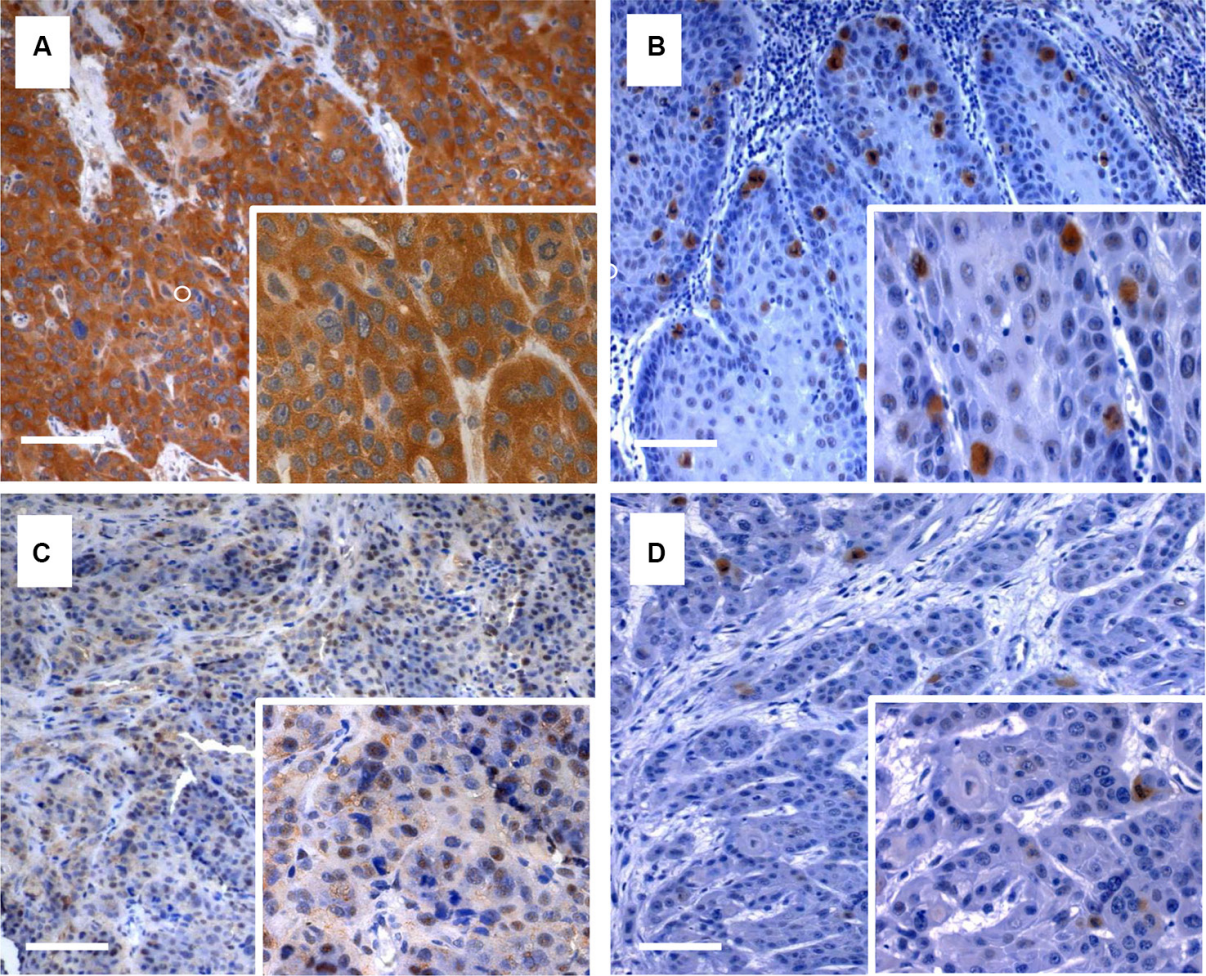
Immunohistochemical staining of Plk3 and pT273 caspase-8 Examples of anal cancer biopsies with high (**A**, **C**) and low (**B, D**) immunohistochemical detection of Plk3 and pT273 caspase-8. Original magnification × 100 (inlets × 400), scale bar: 100 μm.

Characteristics of Plk3 expression and levels of pT273 caspase-8 based on the cut off values are given in Table 1. Sixty patients (63.2%) had a high Plk3 expression, fifty patients (52.6%) had high levels of pT273 caspase-8. Representative examples of low and high Plk3 and pT273 caspase-8 immunohistochemical staining are depicted in Figure 1.

**Table 1:** Results of Plk3 and pT273 caspase-8 immunohistochemistry

Marker	Plk3 *n* (%)	pT273Casp 8 *n* (%)
Dichotomized score	≤ 6 WS > 6	≤ median >
low score	34 (35.8)	45 (47.4)
high score	60 (63.2)	50 (52.6)

Dichotomized score low vs. high based on the median weighted score (WS: intensity x % pos. tumor cells), median pT273 Casp8 positive cells (%)

### Patient histopathological characteristics, according to Plk3 expression and phosphorylation of caspase-8 at T273

Patient- and tumor-related characteristics according to Plk3 expression and pT273 caspase-8 levels are shown in Table 2. A low Plk3 detection was more prevalent in patients ≤ 58 years (*p* = 0.015), whereas high Plk3 expression was more common in patients with HPV-16 viral load > median (*p* = 0.033). By contrast, we observed no significant differences for gender, T- and N-category, tumor grading and total dose including boost. pT273 caspase-8 signals were significantly higher in female patients (*p* = 0.02), in T1/2 tumors (*p* = 0.014) and in those with high HPV-16 DNA load (*p* = 0.006) and p16^INK4a^ expression (*p* = 0.003). Finally, high Plk3 expression was significantly more prevalent in patients with high levels of pT273 caspase-8 (*p* < 0.001) and vice versa, indicating a highly close correlation between these molecular markers (Table 2).

**Table 2:** Clinicopathological findings according to Plk3 expression and pT273 Caspase-8 levels

	**No. of patients**	**Plk3 WS ≤ 6 *n* (%)**	**PLK3 WS > 6 *n* (%)**	***p*-value**	**No. of patients**	**Casp8 ≤ Med *n* (%)**	**Casp8 > Med *n* (%)**	***p*-value**
**Age** ≤ 58 years > 58 years	48 46	23 (67.6) 11 (32.4)	25 (41.6) 35 (58.4)	**0.015**	49 46	26 (56.5) 20 (43.5)	24 (48.0) 26 (56.0)	0.46
**Gender** male female	40 54	17 (50.0) 17 (50.0)	23 (38.3) 37 (61.7)	0.27	41 54	25 (55.5) 20 (43.5)	16 (32.0) 34 (68.0)	**0.02**
**T-stage** T1/2 T3/4 Tx	67 26	20 (60.6) 13 (39.4)	47 (78.3) 13 (21.6)	0.07	67 27 1	26 (57.7) 18 (40.0) 1 (2.3)	41 (82.0) 9 (18.0)	**0.014**
**N-stage** N0 N1-3 Nx	61 27 6	21 (61.8) 10 (29.4) 3 (8.8)	40 (66.6) 17 (28.4) 3 (5.0)	0.81	61 28 6	25 (55.5) 16 (35.5) 4 (8.0)	36 (72.0) 12 (24.0) 2 (4.0)	0.15
**Grading** G1/2 G3	76 18	26 (76.4) 8 (23.6)	50 (83.3) 10 (16.7)	0.42	77 18	37 (82.3) 8 (17.7)	40 (80.0) 10 (20.0)	0.78
**Total dose** (including boost) ≤ 50.4 Gy > 50.4 Gy	56 38	19 (55.9) 15 (44.1)	37 (61.7) 23 (38.3)	0.664	57 38	30 (66.7) 15 (33.3)	27 (54.0) 23 (46.0)	0.294
**HPV-16 load** HPV-16 Med HPV-16 > Med	31 49	20 (50.0) 20 (50.0)	11 (28,9) 29 (71.1)	**0.033**	36 44	24 (60.0) 16 (40.0)	12 (30.0) 28 (70.0)	**0.006**
**p16^INK4A^** p16 WS ≤ 6 p16 WS > 6	22 72	7 (20.5) 27 (79.5)	15 (27.7) 45 (72.3)	0.063	33 62	17 (37.8) 28 (62.2)	6 (12.0) 44 (88.0)	0.003
**HIV-status** positive negative	17 76	9 (27.3) 24 (72.7)	8 (13.3) 52 (86.7)	0.09	17 77	10 (22.7) 34 (77.3)	7 (14.0) 43 (86.0)	0.27
**Plk3** Plk3 WS ≤ 6 Plk3 WS > 6					34 60	30 (68.2) 14 (31.8)	4 (8.0) 46 (92.0)	**< 0.001**
**pT273 Casp8** Casp8 ≤ Med Casp8 > Med	44 50	30 (88.2) 4 (11.8)	14 (23.3) 46 (76.6)	**< 0.0001**				

### Univariate and multivariate analyses of oncologic outcomes

After initial treatment, clinical complete response was documented in 83 of 95 patients (87.4%) at the first re-staging examination while 10 patients (10.5%) developed either locoregional progressive disease (*n* = 3) or distant metastases (*n* = 7). From these patients, seven underwent palliative chemotherapy or best supportive care, and three patients received salvage surgery (R0-resection in two, R1-resection in one). One patient died after completion of CRT due to pneumonia, and one was lost to follow-up before re-staging.

**Figure 2:**
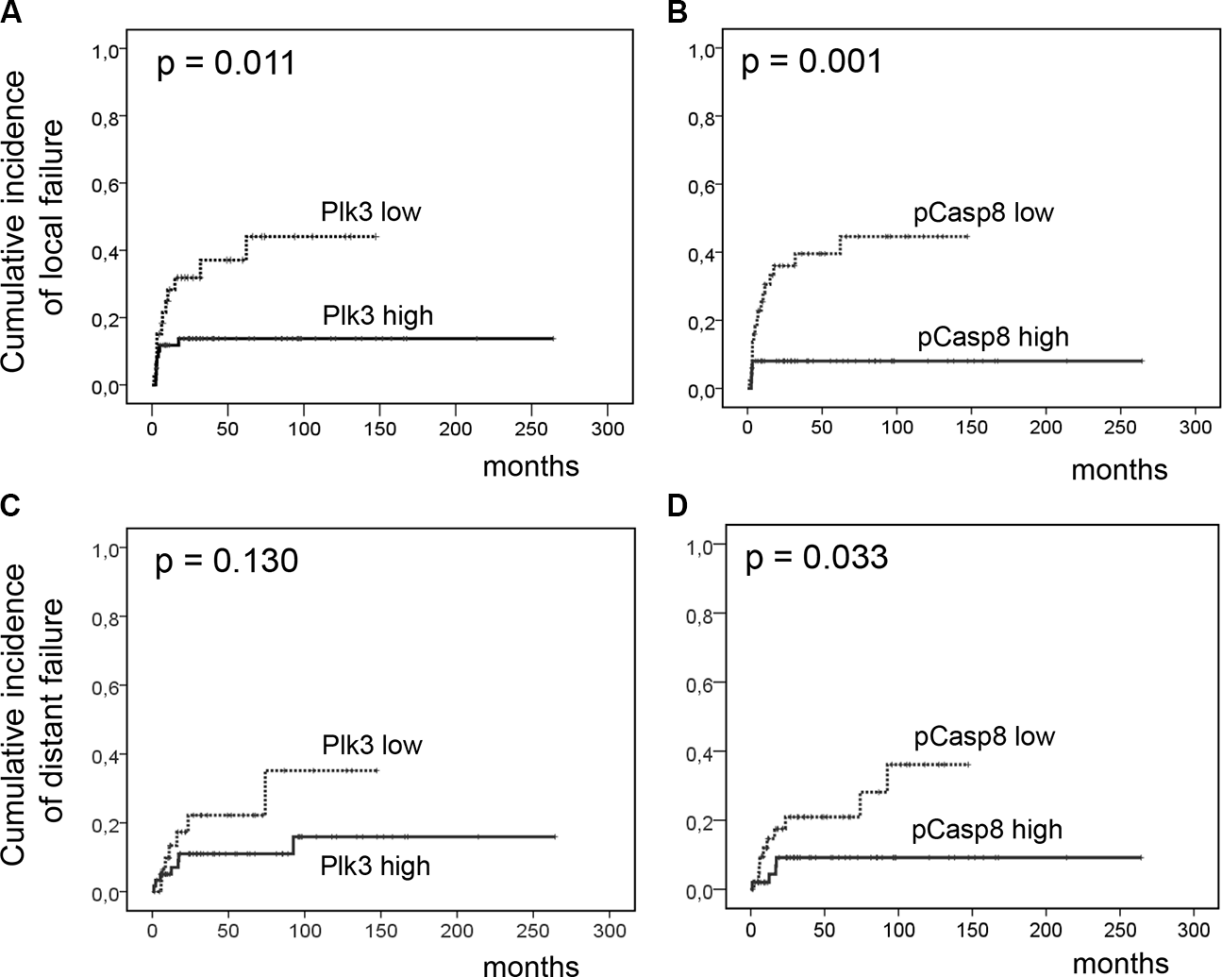
Incidence of locoregional and distant failure according to Plk3 and pT273 caspase-8 expression Cumulative incidence of locoregional (**A**–**B**) and distant failure (**C**–**D**) according to low Plk3 expression (individual WS ≤ 6) and pT273 caspase-8 levels (≤ median) vs. high Plk3 expression (WS > 6) and high pT273 caspase-8 levels (> median) in pretreatment biopsies of patients with anal carcinoma treated with definitive CRT.

Further follow-up in 83 patients with initial complete response revealed a locoregional recurrence in 9 patients and distant metastases in 14 patients (14.7%). A total of 27 patients died during follow-up: 16 of anal cancer, and 11 of intercurrent diseases. Patients with low Plk3 expression (Figure 2A) and low pT273 caspase-8 levels (Figure 2B) had significantly worse local failure rates (*p* = 0.011, *p* = 0.001) compared to patients with a high Plk3 and pT273 caspase-8 detection. Additional clinicopathologic factors with a significant impact on local failure rates in univariate analysis included T-stage (*p* = 0.001) and N-category (*p* < 0.001). In multivariate analyses, N-stage (*p* = 0.003) and Plk3 expression (*p* = 0.018) remained significant independent factors (Table 3) for local failure. The cumulative incidence of distant metastases for the entire group was 14.5% and 21.1% at 5 and 10 years, respectively. T/N category and pT273 caspase-8 levels were significantly related to the risk to develop distant metastases in univariate analysis (*p* = 0.002 and *p* = 0.01, Figure 2C), while Plk3 expression did not reach a level of significance (*p* = 0.13 Figure 2D). In multivariate analysis, T-stage remained a significant predictor for distant metastases (*p* = 0.026, Table 3).

**Table 3:** Univariate and multivariate analyses of prognostic factors in patients with anal SCC

				Multivariate	
			95% CI		
	Univariate *p*-value	HR	lower	upper	*p*-value
**Cumulative incidence of local failure**					
T-stage (T3-4/T1-2)	**0.001**	1.70	0.46	6.28	0.41
N-stage (N1-3/N0)	**< 0.001**	5.43	1.80	16.35	**0.003**
HPV-16 load (≤/> median)	**0.022**	2.94	0.81	10.71	0.10
p16^INK4a^ (WS ≤ 6/> 6)	**0.021**	1.07	0.32	3.55	0.90
Plk3 (WS ≤ 6/> 6)	**0.011**	4.18	1.27	13.71	**0.018**
Casp8 (≤/> median)	**0.001**	1.55	0.38	6.26	0.53
**Cumulative incidence of distant failure**					
T-stage (T 3–4/T 1–2)	**0.002**	4.05	1.18	13.84	**0.026**
N-stage (N 1–3/N 0)	**0.01**	2.19	0.610	7.87	0.22
Casp8 (≤/> median)	**0.033**	2.90	0.76	11.11	0.11
**Cancer-specific survival**					
T-stage (T 3–4/T 1–2)	**0.001**	2.57	0.69	9.57	0.16
N-stage (N 1–3/N 0)	**< 0.001**	9.94	2.95	33.55	**0.001**
Plk3 (WS ≤ 6/> 6)	**0.011**	3.94	1.29	11.90	**0.016**
Casp8 (≤/> median)	**0.013**	1.34	0.34	5.32	0.67
**Overall survival**					
T-stage (T 3–4/T 1–2)	**0.008**	1.05	0.38	2.86	0.93
N-stage (N 1–3/N 0)	**0.002**	3.96	1.61	9.72	**0.003**
HPV-16 load (≤/> median)	**0.020**	2.19	0.87	5.50	0.095
Plk3 (WS ≤ 6/> 6)	**0.024**	2.97	1.16	7.58	**0.023**
Casp8 (≤/> median)	**0.001**	2.46	0.53	4.89	0.41

**Figure 3:**
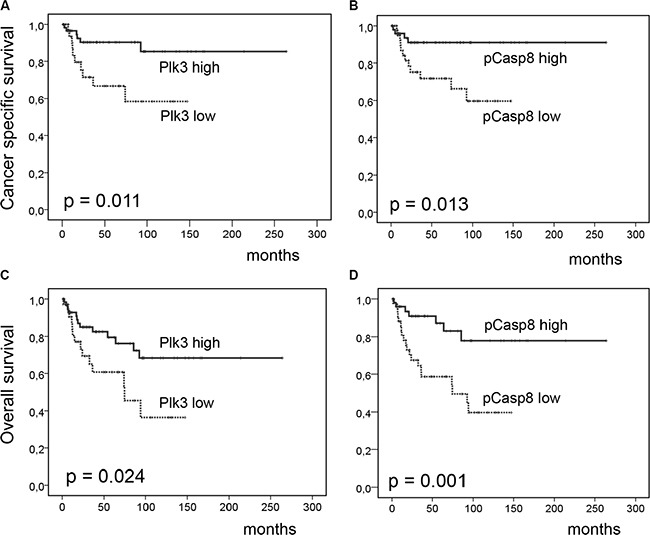
Cancer-specific and overall survival according to Plk3 and pT273 caspase-8 expression Cancer-specific (**A**–**B**) and overall survival (**C**–**D**) according to low Plk3 detection (WS ≤ 6) and low pT273 caspase-8 levels (≤ median) vs. high Plk3 detection (WS > 6) and high pT273 caspase-8 Levels (> median) in pretreatment biopsies of patients with anal carcinoma treated with definitive CRT.

As depicted in Figure 3A and 3B, Plk3 expression (*p* = 0.011) and pT273 caspase-8 levels (*p* = 0.013) were significantly related to CSS in univariate analyses. Again, Plk3 expression retained significance (*p* = 0.016) in multivariate analyses (Table 3). Furthermore, for OS (Figure 3D), we observed a significant correlation to Plk3 and pT273 caspase-8 immunoreactivity (*p* = 0.024 and *p* = 0.001) in univariate analyses (Table 3), while N-stage, and Plk3 expression remained independent significant prognostic factors (*p* = 0.003, and *p* = 0.023) in multivariate analyses for this parameter (Table 3).

### Correlation of Plk3 and caspase-8 with HPV16 DNA-load

**Figure 4:**
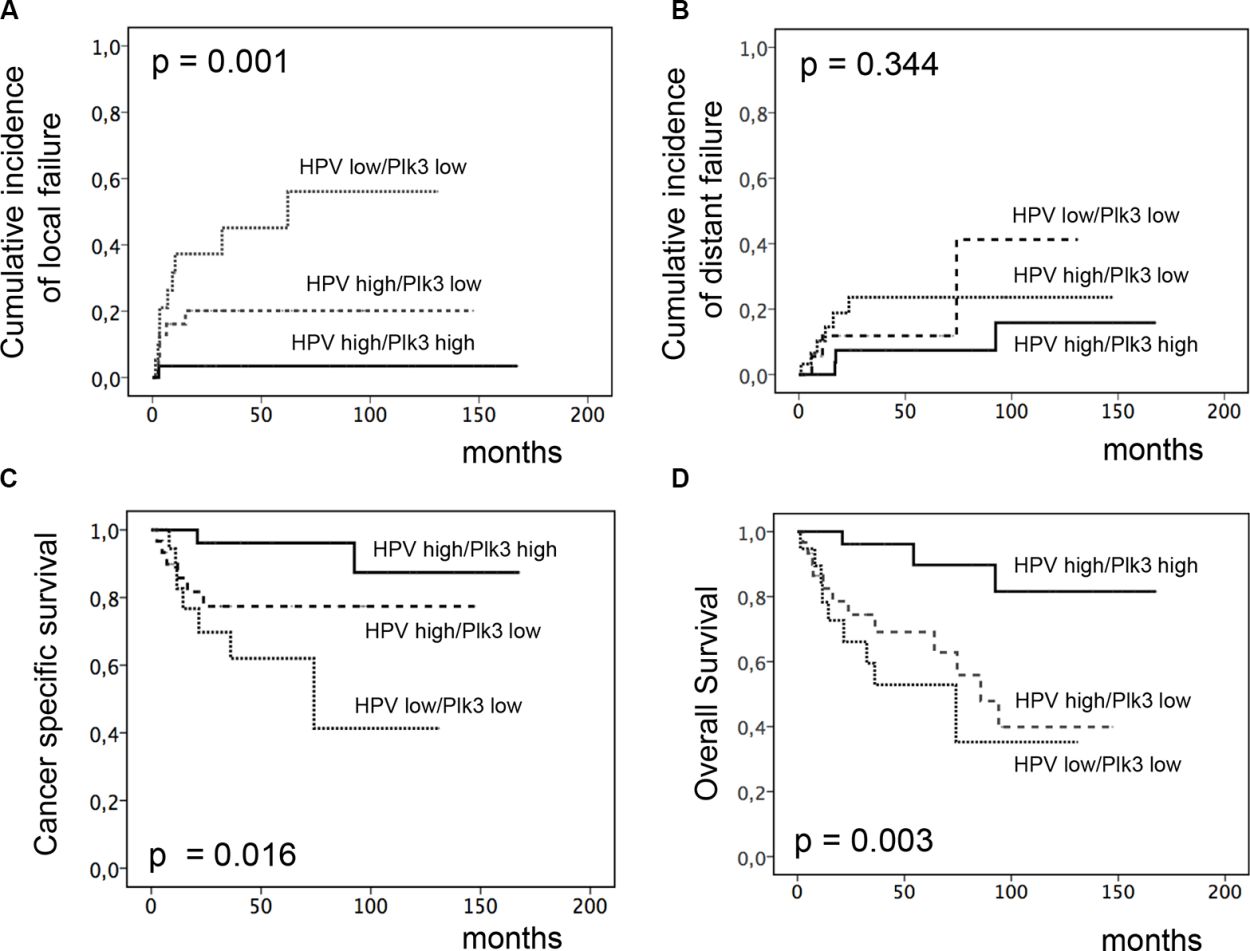
Incidence of locoregional and distant failure, CSS and OS according to combined HPV16 DNA load and Plk3 expression Cumulative incidence of locoregional. (**A**) and distant failure (**B**), CSS (**C**) and OS (**D**) according to combined HPV16 DNA load and Plk3 detection (high HPV16 DNA load and high Plk3 vs. high HPV16 DNA load and low Plk3 vs. low HPV16 DNA load and low Plk3 expression) in pretreatment biopsies of patients with anal carcinoma treated with definitive CRT.

**Figure 5:**
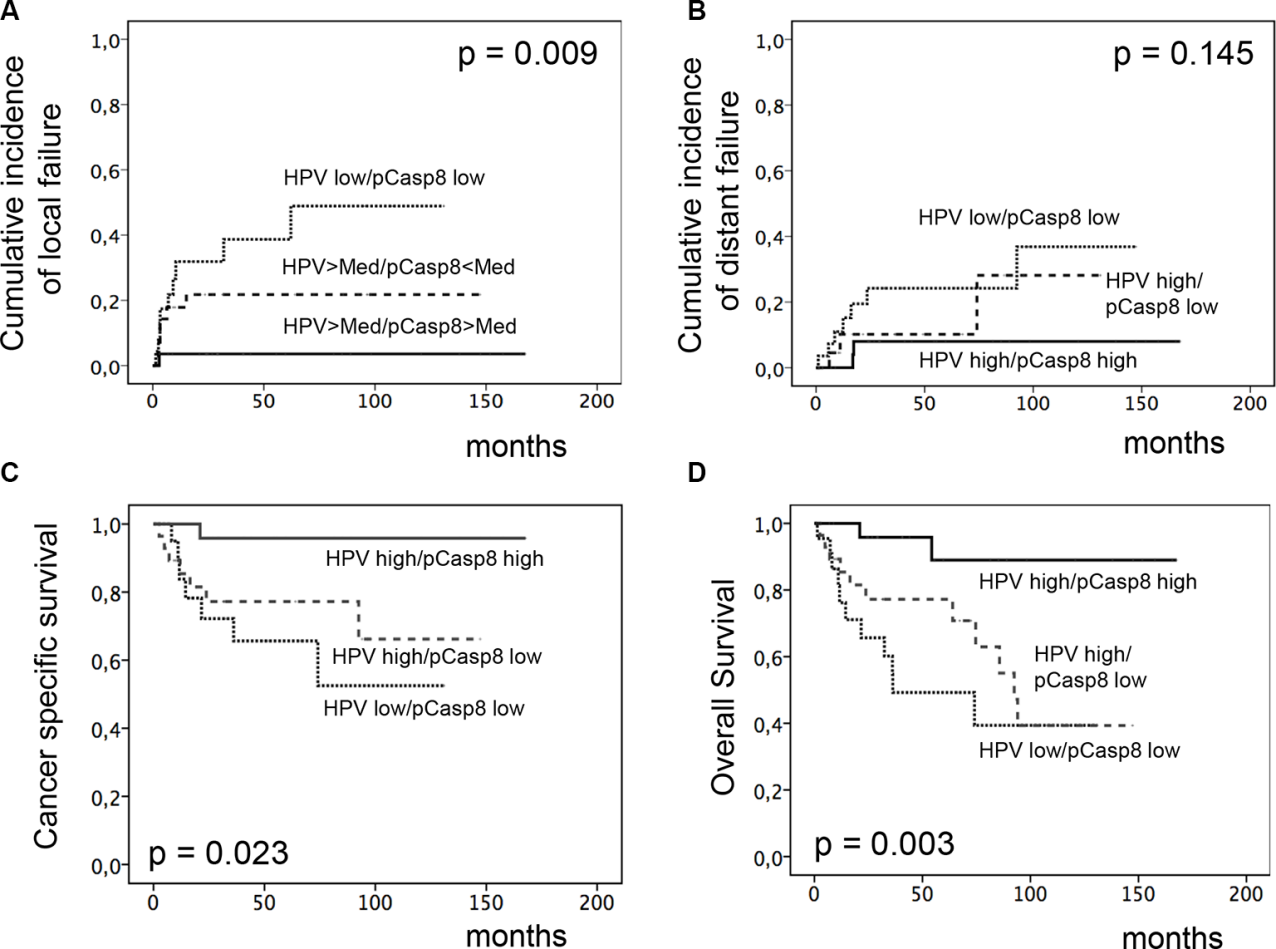
Incidence of locoregional and distant failure, CSS and OS according to combined HPV16 DNA load and pT273 caspase-8 expression Cumulative incidence of locoregional. (**A**) and distant failure (**B**), CSS (**C**) and OS (**D**) according to combined HPV16 DNA load and pT273 caspase-8 levels (high HPV16 DNA load and high pT273 caspase-8 vs. high HPV16 DNA load and low pT273 caspase-8 vs. low HPV16 DNA load and low pT273 caspase-8) in pretreatment biopsies of patients with anal carcinoma treated with definitive CRT.

We have previously shown that elevated levels of HPV-16 viral DNA load and surrogate marker p16^INK4a^ expression predict for improved local tumor control and OS in the patients cohort analyzed for Plk3 and pT273 caspase-8 levels in the present study [22]. Thus, we asked for an interrelationship between these molecular markers, that is shown by significant correlations most pronounced for Plk3 (*p* = 0.033) and pT273 caspase-8 (*p* = 0.006) with HPV16 DNA load (Table 2). Moreover, we performed analyses on combined marker variables. As shown in Figure 4A combined high HPV16 DNA load (> median) and high Plk3(> WS 6) variable was significantly related to decreased local failure (*p* = 0.001, and increased CSS (*p* = 0.016) and OS (*p* = 0.003) and remained significant in multivariate analyses for local failure and OS (Supplementary Table 1). The same holds true for a combined HPV-16 DNA load and pT273 caspase-8 variable (Figure 5) with *p* = 0.009 (local failure), *p* = 0.023 (CSS), and *p* = 0.003 (OS). Finally, a combined p16^INK4a^ and Plk3 (Supplementary Figure 1) or p16^INK4a^ and pT273 caspase-8 variable (Supplementary Figure 2) revealed a significant relationship to local failure (*p* = 0.006, *p* = 0.004), CSS (*p* = 0.004, *p* = 0.030) and OS (*p* = 0.001, *p* = 0.016).

## DISCUSSION

While the role of Plk1, the prototype member of the Plk family as a diagnostic marker and molecular target, has been extensively analyzed in a multitude of malignancies [2, 5], the role of Plk3 seems to differ in various tumor entities [3]. In hepatocellular carcinoma, a weak mRNA and protein expression correlated with shorter patient survival due to promoter hypermethylation or loss of heterozygosity at the Plk3 loci [18]. Additional studies reported overexpression of Plk3 to be significantly associated with adverse clinical outcome in ovarian [20] and breast cancer [21]. To the best of our knowledge, however, the impact of Plk3 expression levels on prognosis and treatment response in anal SCC has not been analyzed so far. In the present study, we showed that high expression of Plk3 correlated with increased local tumor control (Figure 2) and a long-term survival benefit (Figure 3) in a cohort of 95 patients treated homogeneously by concomitant CRT. Although the exact molecular basics of Plk3's role in prolonging patient survival following RCT remains unclear, it is likely related to DNA damage and apoptosis induction [3, 11, 14]. Plk3-mediated S20 phosphorylation of TP53 promotes its stability following DNA damage [11], while induction of Plk3 transcription in an Ataxia telangiectasia mutated (ATM) kinase- and TP53-dependent manner indicates a reciprocal regulatory mechanism for TP53 signal amplification [23]. Interaction with Chk2 [24] and induction by transcription factor RelA-nuclear factor κB (NF-κB) activity [25] further mediates pro-apoptotic signaling by Plk3. Most recently, we demonstrated that Plk3 interacts with the death receptor CD95 promoting apoptosis (14).

Although data on a prognostic impact of pre-therapeutic apoptosis in anal SCC are rare [26], there is emerging evidence that elevated levels of apoptosis may display a significant prognosticator for lower rates of local tumor recurrences and increased survival in intestinal tumors treated with RCT [27, 28]. Caspase-8 was first identified as a critical effector of death receptor mediated cell killing but also modulates adhesion to the extracellular matrix, activation of the Erk kinase cascade and cell mobility [29–32]. Furthermore, caspase-8 has been recognized as novel substrate of Plk3 as phosphorylation of the protein at residue T273 enhanced its pro-apoptotic function [15]. In that context, we here demonstrate a highly significant correlation between Plk3 and pT273 caspase-8 detection (Table 2) and thus phosphorylation of caspase 8 may represent a mechanism by which different levels of Plk3 expression in tumor cells may stimulate the extrinsic apoptotic machinery. Given the intense selection pressure present in a tumor's microenvironment, upregulation of pT273 caspase-8 in some tumors may also affect the metastatic propensity of cancer cells. Importantly, we here describe elevated levels of pT273 caspase-8 to be significantly correlated with a reduced incidence of distant metastases (Figure 2E) confirming this assumption.

In HPV-associated malignancies, particularly in head and neck (HNSCC) and anal squamous cell carcinoma, most studies revealed a more favorable prognosis in patients with an HPV-positive tumor compared to HPV-negative patients. Moreover, response towards RT or CRT is superior in HPV-carrying tumors indicating an increased RT/CRT sensitivity in these tumor cells [22, 33–35]. Underlying mechanisms are multifactorial and may include elevated levels of residual DNA double-strand breaks (DSBs) [36], increased radiation-induced G2 cell cycle arrest [37] and modulation of AKT activation [38]. Moreover, a differential response further attributes to distinct genomic landscapes of the individual tumors with phosphoinositol-3-kinase pathway (PI3K/AKT/mTOR) gene alterations including amplifications and homo- zygous deletions present in 63% of cases [38–40]. Additionally, tumor suppressor gene TP53 alterations were sporadically detected in HPV-positive cells while disruptive mutations very frequently occur in HPV-negative ones [41, 42] implying that TP53 protein degradation by the viral oncoprotein E6 may supersede p53 mutations in HPV-positive tumors. Importantly, Kimple et al. further reported on an increased expression of (wildtype) TP53 and associated genes in HPV carrying cells following irradiation [35] indicating that low levels of remaining TP53 could be activated in these cells resulting in enhanced apoptosis. Accordingly, one may speculate that an improved local tumor control in HPV-expressing anal carcinoma may be associated with these mechanisms including apoptosis regulation by Plk3 and T273 phosphorylation of caspase 8. Indeed, our findings indicate a prominent local effect of both markers in response to RCT (Figure 2), and a significant correlation between Plk3 and pT273 caspase-8 levels and HPV16 viral load or surrogate marker p16^INK4a^ expression (Table 2). Moreover, combined HPV16 DNA load/p16^INK4a^ and especially Plk3 variables revealed an increased significance in predicting local recurrence, CSS and OS (Figures 4–5 and Supplementary Figure S1). Consequently, as the prevalence of HPV-DNA in anal carcinoma commonly exceeds 90% [22, 34], our data on the interrelationship between HPV16 DNA load/p16^INK4a^ immunostaining and Plk3 or pT273 caspase-8 expression may provide additional informations to a histopathological assessment. Notably, HPV viral oncogenes, most important E2, E5, E6 and E7, further interfere with apoptotic pathways via multiple regulatory principles. While HPV-E2 protein induces apoptosis via downregulating the transcription of E6/E7 mRNA, HPV-E5 and HPV-E6 prevent hosttriggered apoptosis by impairing CD95 and TRAIL receptor activation (E5), and by promoting amongst others proteasomal degradation of TP53 (E6). HPV-E7 displays both anti- and pro-apoptotic properties including inhibition of a retinoblastoma protein/E2F complex and initiation of TRAIL and TNFα mediated death receptors activation [43, 44]. Even though there is no direct experimental evidence at present in anal carcinoma, one may draw the conclusion by analogy that an increased local control probability and survival in patients with a high Plk3 expression/pT273 caspase-8 levels and a high HPV16 load may be attributed to an interrelationship of these mechanisms.

Our findings further indicate a prominent local effect of Plk3 expression and pT273 caspase 8 phosphorylation in response to RCT as exemplified by a significant impact on local tumor control (Figure 2). In line with that, there is emerging evidence that elevated levels of tumor infiltrating cluster of differentiation (CD)8-positive cytotoxic T lymphocytes (TILs), which are crucial components of tumor-specific cellular immunity [45], have a favorable effect on prognosis and patient survival in a multitude of cancers, including HNSCC and anal SCC [46–49]. Considering a putative clinical importance of CD8(+) TILs carrying the CD95 ligand [50] to modulate activation of Plk3 and pT273 caspase 8 phosphorylation, future investigations will focus on the level of an intratumoral (local) presence of cytotoxic T-cells, CD95 expression and correlation of these parameters with Plk3 and pT273 caspase 8 marker detection.

We would like to acknowledge the limitations of the study. First, the retrospective nature of the analysis cannot exclude calculation bias. Second, these observations warrant further investigation in an extended patient's cohort and a confirmation of their correlative and functional interrelationship in additional tumor entities.

In conclusion, we here demonstrate that low expression of Plk3 and low levels of pT273 caspase-8 detection may, especially if combined with HPV-16 DNA-load, provide valuable prognostic markers in anal cancer. Importantly, these findings may also have therapeutic implications for the usage of Plk(1) antagonists as a variety of ATP-competitive Plk1 inhibitors in clinical evaluation have been shown to exhibit significant levels of cross-reactivity with Plk3 and to inhibit Plk3 activity [9, 51]. These data suggest a pivotal impact of the propensity to undergo apoptotic pathways on response to RCT and clinical outcome that in conjunction with HPV detection warrant further investigation.

## MATERIALS AND METHODS

### Patient characteristics

A total of 95 patients, treated homogeneously with concomitant CRT for anal SSC at the Departments of Radiotherapy and Oncology at the University Hospital of Frankfurt/Main and at the University Medical Center of Göttingen were included in the study following an institutional review board approval. Eligibility criteria included histological proof of anal SCC, and curative intent of 5-fluorouracil (5-FU)/mitomycin C-based CRT. Before treatment, patients were routinely subjected to physical and rectal-digital examination, proctoscopy with biopsy, CT/MRI of the abdomen and pelvis, chest X-ray, serum chemistry, and complete blood count.

### Treatment modalities and follow-up

Patients were treated by photon beam linear accelerators (Elekta, Crowley, UK; Varian, Palo Alto, USA) using either 3-D conformal RT or intensity-modulated RT (IMRT) with a median dose of 50.4 Gy (range 46.8–64.8 Gy, including the boost) with daily fractions of 1.8–2 Gy. A brachytherapy-boost with a dose of 10 Gy was applied to two patients. Twenty-seven patients received an external boost to the primary tumor and/or enlarged lymph nodes of median 7.2 Gy (range 5.4 – 10.8 Gy). Concurrent chemotherapy was applied in two cycles of 5-FU (1.000 mg/m2/24 hours) as four- or five-day continuous infusion in the first and fifth week of RT; mitomycin C (10 mg/m^2^) was administered as intravenous bolus on day one of each cycle. Treatment response was assessed by rectal-digital examination and proctoscopy (including biopsies taken in case of suspicious residual tumor), and by pelvic CT/MRI-scan 6–8 weeks after completion of therapy. Follow-up examinations, including physical and rectal-digital exploration and proctoscopy were scheduled every three months for the first two years, followed by six months intervals.

### Immunohistochemical staining and scoring for Plk3 and pT273 caspase-8

For staining purposes, pretreatment formalin fixed paraffin embedded (FFPE) biopsy tissue slides were subjected to a staining procedure with DAKO EnVision™ FLEX Peroxidase Blocking reagent (K8000, DAKO, Hamburg, Germany) and primary anti-Plk3 (Abcam, Cambrigde, UK) and anti-pT273 caspase-antibodies [15] were applied at a 1:100 dilution. Next, dextran polymer conjugated horseradish peroxidase and 3,3′-diaminobenzidine (DAB) chromogen was used for visualization and hematoxylin solution for counterstaining. Negative control slides in the absence of primary antibodies were included for each staining procedure. Methods for quantification of PCR-based HPV-16 virus DNA-load and histochemical p16^INK4a^expression were reported in more detail previously [22]. To minimize interobserver variability, two investigators (F.R, P.B) without knowledge of the clinicopathological or clinical data performed scoring. Plk3 immunoreactivity was assessed considering both, the fraction of Plk3 positive tumor cells (1: (0–25%), 2: (26–50%), 3: (51–75%) and 4: (> 75%)) and the intensity of staining scored as 1+ (weak), 2+ (moderate) and 3+ (intense). Next, these parameters were multiplied to produce an individual weighted score (WS). A WS ≤ 6 was defined as “low” and a WS of > 6 as “high” Plk3 expression. Phosphorylation of caspase- 8 at T273 was assessed by dividing the number of positive tumor cells by the total number of tumor cells, multiplied by 100.

### Statistical analysis

Assessment of the correlation between Plk3 expression and pT273 caspase-8 levels was performed using the Spearman's correlation coefficient. The cumulative incidence of locoregional failure and distant metastasis were defined as the time from start of CRT to the first locoregional tumor detection (i.e. persistent tumor at re-staging, any regional tumor recurrence after initial complete response) or detection of distant metastases. Data from patients who were alive and free of recurrences or who died without having a recurrence were censored for these endpoints. Overall (OS) and cancer specific survival (CSS) were defined as the time of start of CRT to death for any reasons or to cancer-related death, or the day of the last follow-up. Survival was plotted according to the Kaplan-Meier method, univariate and multivariate analyses were performed using the log-rank test and the Cox proportional hazard model, respectively. A *p* < 0.05 was considered statistically significant in all testing. IBM SPSS Version 21 was used for all statistical analyses.

## SUPPLEMENTARY MATERIALS FIGURES AND TABLE


