# Supplementary material for: Polo-like kinase 3 and phosphoT273 caspase-8 are associated with improved local tumor control and survival in patients with anal carcinoma treated with concomitant chemoradiotherapy
Source: Oncotarget. 2016 Jul 23;7(33):53339–49. doi: 10.18632/oncotarget.10801 (PMC5288191; doi:10.18632/oncotarget.10801)
Supplement: Supplementary file 1 [file oncotarget-07-53339-s001.pdf]

Polo-like kinase 3 and phosphoT273 caspase-8 are associated with improved local tumor control and survival in patients with anal carcinoma treated with concomitant chemoradiotherapy

Supplementary Materials

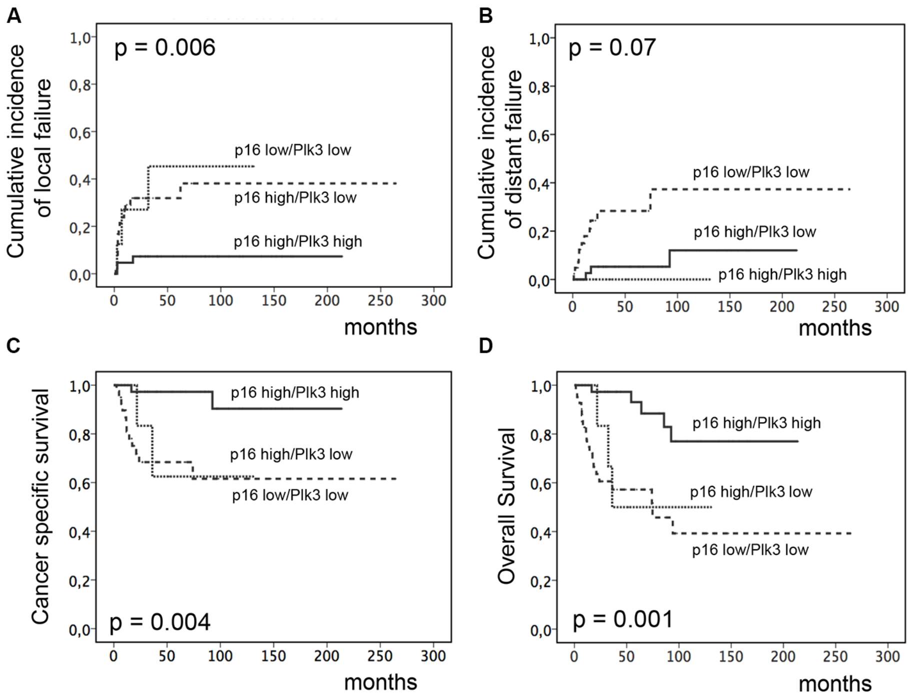

Supplementary Figure S1: Incidence of locoregional and distant failure, CSS and OS according to combined p16INK4a and Plk3 expression. Cumulative incidence of locoregional (**A**) and distant failure (**B**), CSS (**C**) and OS (**D**) according to combined p16^{INK4a} and Plk3 expression (high Plk3 and high p16^{INK4a} vs. high Plk3 and low vs. low Plk3 and low p16^{INK4a}) in pretreatment biopsies of patients with anal carcinoma treated with definitive CRT.

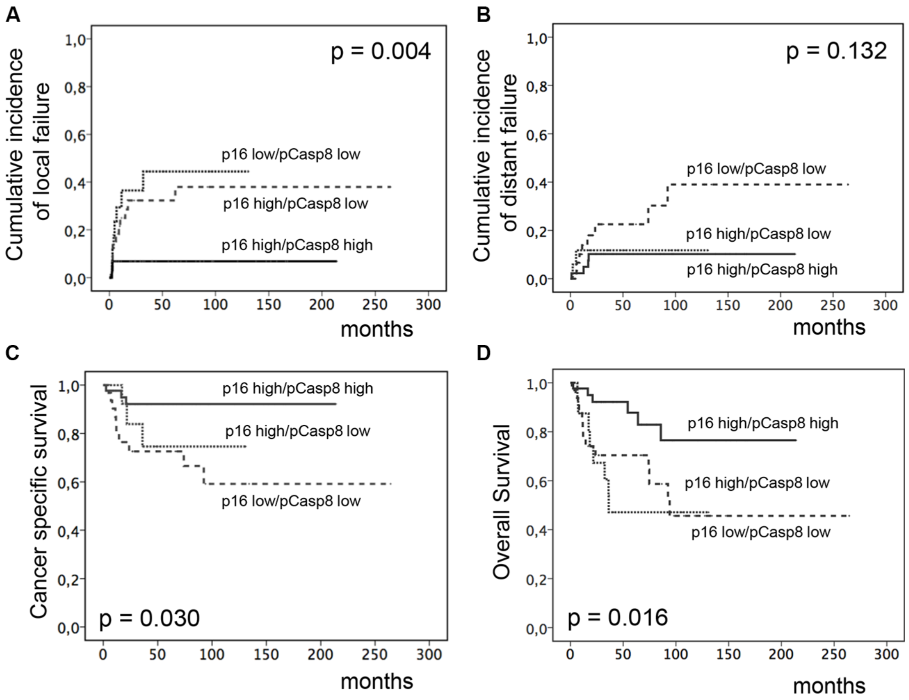

Supplementary Figure S2: Incidence of locoregional and distant failure, CSS and OS according to combined p16^{INK4a} and pT273 caspase-8 expression. Cumulative incidence of locoregional (**A**) and distant failure (**B**), CSS (C) and OS (**D**) according to combined p16^{INK4a} and pT273 caspase-8 levels (high pT273 caspase-8 and high p16^{INK4a} vs. high pT273 caspase-8 and low p16^{INK4a} vs. low pT273 caspase-8 and low p16^{INK4a}) in pretreatment biopsies of patients with anal carcinoma treated with definitive CRT.

Supplementary Table S1: Univariate and multivariate analyses of prognostic factors and combined HPV16 DNA load/p16^{INK4a} and Plk3 or HPV16 DNA load/p16^{INK4a} and pT273 Caspase 8 variable in patients with anal SCC

		Multivariate			
			95 % CI		
	**Univariate *p*-value**	**HR**	**lower**	**upper**	***p*-value**
**Cumulative incidence of local failure**					
N-stage (N1-3/N0)	**< 0.001**	6.15	1.90	19.84	**0.002**
combined HPV16 load/Plk3[a]	**0.001**	16.77	2.09	134.0	**0.008**
combined HPV16 load/pT273	**0.009**	11.18	0.72	171.66	0.083
combined p16^{INK4a}/Plk3	**0.006**	5.80	0.94	35.65	0.058
combined p16^{INK4a}/pT273	**0.004**	3.33	0.77	14.33	0.106
**Cancer-specific survival**					
N-stage (N 1-3/N 0)	**< 0.001**	6.72	2.03	22.26	**0.002**
combined HPV16 load/Plk3	**0.016**	10.21	1.18	55.36	**0.007**
combined HPV16 load/pT273	**0.023**	2.58	0.20	32.94	0.605
combined p16I^{NK4a}/Plk3	**0.004**	7.22	1.53	34.00	**0.012**
combined p16I^{NK4a}/pT273	**0.030**	2.19	0.62	7.68	0.221
**Overall survival**					
N-stage (N 1-3/N 0)	**0.002**	4.30	1.77	10.42	**0.05**
combined HPV16 load/Plk3	**0.003**	9.18	2.28	36.97	**0.002**
combined HPV16 load/pT273	**0.003**	3.44	0.58	20.28	0.171
combined p16^{INK4a}/Plk3	**0.004**	5.08	1.80	14.31	**0.002**
combined p16^{INK4a}/pT273	**0.001**	2.14	0.67	6.83	0.179
